# Aging Alters mRNA Processing in the Mouse Ovary

**DOI:** 10.3390/cells14130996

**Published:** 2025-06-30

**Authors:** Kevin Vo, Grace J. Pei, Ramkumar Thiyagarajan, Patrick E. Fields, M. A. Karim Rumi

**Affiliations:** 1 Pathology and Laboratory Medicine, University of Kansas Medical Center, Kansas City, KS 66160, USA; kvo5@kumc.edu (K.V.); 3096823@smsd.org (G.J.P.); pfields@kumc.edu (P.E.F.); 2 Internal Medicine (GERI), University of Kansas Medical Center, Kansas City, KS 66160, USA; rthiyagarajan@kumc.edu

**Keywords:** ovary, aging, gene expression, pre-mRNA processing, transcript variants, RNA binding proteins

## Abstract

Aging in females affects the ovaries before any other organ. This has a significant impact on women’s health. Aging results in the gradual depletion of ovarian follicles and a decline in oocyte quality. Studies have shown that cellular changes within ovaries manifest before the depletion of ovarian follicles. To understand the molecular mechanisms underlying these changes, we conducted a comprehensive analysis of gene expression changes in aging mouse ovaries. When RNA sequencing data from 6-month-old mice were compared to those from 12-month-old mice, we identified numerous differentially expressed genes, as well as transcript variants. Transcript variants arise from alternative transcription start sites (TSSs) and alternative pre-mRNA processing. Therefore, we further analyzed a specific set of regulators for these cellular processes. Our findings indicate that ovarian aging alters the expression of epigenetic regulators (ERs) and transcription factors (TFs) that are involved in alternative TSS usage. Ovarian aging also affects the expression of RNA-binding proteins (RBPs) and spliceosome components (SPs), which are essential for pre-mRNA processing. We noticed that variations in transcript variants were more pronounced than those found through gene expression analysis. While 8% of the known TFs and ERs were differentially expressed at the gene level, this increased to 30% at the transcript variant level. Similarly, 3% of the known RBPs but no known SPs were differentially expressed at the gene level, while this increased to 30% at the transcript variant level. These observations highlight the importance of focusing on transcript variants and their functions in aging research, as they may provide insight into the underlying biological processes involved.

## 1. Introduction

Ovarian aging is a poorly understood natural process of declining ovarian steroidogenesis and oogenesis [1]. This impacts all women over 45 years old and remains a significant barrier to improving women’s health. The loss of ovarian follicles is the primary cause of ovarian aging [2]. However, age-induced disruption in the hypothalamic–pituitary axis and changes in cellular functions also occur during ovarian aging [3,4]. Several factors accelerate ovarian aging, such as oxidative stress, cohesion deterioration, cellular senescence, gene mutations, autoimmunity, chemotherapy, and radiotherapy [1,5,6,7]. These pathological conditions can also result in premature ovarian insufficiency (POI) in younger women [8,9,10].

It has been reported that cellular changes occur during ovarian aging before the depletion of ovarian follicles. To gain insight into these cellular changes, we analyzed changes in gene expression in aging mouse ovaries. Changes in cellular function depend on changes in gene expression. Accordingly, studies have focused on the differentially expressed genes to understand the mechanisms of ovarian aging. However, we have previously shown that analyses of mRNA transcript variants provide a more accurate representation of differential gene expression. In this study, we have focused on the differential expression of mRNA transcript variants during ovarian aging.

Gene expression is regulated at the epigenetic, transcriptional, and post-transcriptional levels [11,12]. Epigenetic regulators (ERs) and transcriptional factors (TFs) are intricately linked to maintain the dynamic changes in the cellular microenvironment [13]. In general, ERs influence chromatin structure and TF binding, enabling the activation or repression of transcription [14]. ERs and TFs also determine the transcription start site of pre-mRNAs in a cell. Since ERs and TFs are recruited to facilitate these mechanisms, the transcripts of these proteins play a crucial role in the response to pathological conditions, such as oxidative stress and cellular senescence [10]. One of the primary modifications induced by epigenetics is DNA methylation [15]. Reproductive aging induces dynamic alterations in methylation, causing high levels of methylation in the DNA of mature oocytes [16]. These high levels of DNA methylation regulate gene expression, leading to changes in tissue-specific functions, and likely play a role in the aging process. Over time, these oocytes decrease in quantity. This lower number of oocytes possesses decreased levels of DNA methylation. This reduced level of methylation may reflect either a decrease in DNA methylation or an increase in demethylation as the oocytes age [17].

Other mechanisms that can generate mRNA transcript variants occur by the addition of post-transcriptional modifications (PTMs). PTMs are changes made to pre-mRNA molecules after they are transcribed. Known modifications include 5′ capping, polyadenylation, and intron splicing. PTMs of pre-mRNAs are not only essential for splicing and polyadenylation, but also for regulating the translation of proteins [18]. These modifications involve pre-mRNA processing by RNA-binding proteins (RBPs), including spliceosome components (SPs) [19,20]. It has been reported that RBPs can regulate mRNA turnover and translation, which impact age-dependent gene expression [21]. A recent study has reported differing levels of RBPs in human fibroblasts during replicative senescence and in subjects of varying ages [22]. One of these RBPs, AUF1, was detectable in the ovary. Another factor in the splicing of pre-mRNAs is the spliceosome, which is comprised of several small nuclear ribonucleoproteins [23]. During alternative splicing events, altered expression of the spliceosome seems to have an impact on oocyte maturation in human ovaries [24]. Since maturation deficiencies are a significant marker of ovarian aging, new methods that focus on the detection of the mechanisms responsible for the detrimental effects of aging need to be recognized [25].

This study compared the genes and transcript variants expressed in the ovaries of young (6-month-old) and aged (12-month-old) mice to identify differentially expressed genes (DEGs) and differentially expressed transcript variants (DETVs). We identified many DEGs as well as DETVs in aged ovaries, including those that were switched on (not expressed in young ovaries) and those that were switched off (not expressed in aged ovaries). Remarkably, some of the DEGs and DETVs in aged ovaries were reported to be associated with the loss of ovarian reserve.

## 2. Materials and Methods

### 2.1. Sample Collection and Processing

To analyze age-induced changes in gene expression in the ovaries, this study included mice of 6 and 12 months of age. The 6-month-old group represented ovaries before senescence, and the 12-month-old group represented ovaries after a significant decline in follicle reserve [26]. Daily vaginal smears were examined to determine the estrus cycles, and on the day of estrus, mice were euthanized to collect their ovaries. One ovary from each mouse was fixed in 4% formaldehyde and paraffin-embedded, and then the histological sections were stained with hematoxylin and eosin. Histological observations were made using a Nikon 80i bright-field microscope (Nikon Instruments Inc., Melville, NY, USA), and images were captured with a Nikon digital sight DS-Fi3 8-bit camera. The other ovary was snap-frozen in liquid nitrogen and stored at −80 °C until the total RNAs were extracted. All experimental procedures were performed following protocols approved by the Animal Care and Use Committee of the University of Kansas Medical Center or as described in a previous study [26].

### 2.2. RNA Sequencing Data

This study included RNA-Seq data from 6-month-old (young) and 12-month-old (aged) mouse ovaries (*n* = 6). The RNA-Seq data were downloaded from the Sequence Read Archive (SRA) at NCBI (PRJNA1195555) and analyzed as described below. The RNA-Seq data were generated by the Stout laboratory at the Oklahoma Medical Research Foundation [26]. An ovary was collected from each of the 6 mice within the respective age group. Total RNA was extracted using TRIzol reagent (ThermoFisher Scientific, Carlsbad, CA, USA), and the RNA-Seq libraries were prepared using the NEB Ultra II Directional kit (New England Biolabs, Ipswich, MA, USA) [26]. The libraries were sequenced on an Illumina NovaSeq 600 system [26].

### 2.3. RNA Sequencing Analysis

We analyzed the RNA-Seq data from young and old mouse ovaries using CLC Genomics Workbench (GW) 24 (Qiagen Bioinformatics, Redwood City, CA, USA). CLC GW uses the expectation-maximization (EM) estimation algorithm to categorize and assign annotated transcripts to the transcript variants within the reference genome, gene, and mRNA. All clean reads were obtained by removing low-quality reads and trimming the adapter sequences. The high-quality clean reads were aligned to the *Mus musculus* reference genome (GRCm39), gene (GRCm39.113_Gene), and mRNA sequences (GRCm39.113_mRNA) using the default parameters. In addition, we selectively analyzed 2779 TFs, curated by the Gifford lab from a list of human TFs, 651 ERs from a gene atlas, 1296 annotated RBPs, and 504 SPs from two lists of spliceosomes [23,27,28,29,30]. New tracks containing only the specified groups were generated from each RNA-Seq data file that contained GE or TE values, which were then used in subsequent analyses. The expression values of individual genes (GE) or mRNA transcript variants (TE) were measured in transcripts per million (TPM) [24,25,26]. The threshold *p*-value was determined according to the false discovery rate (FDR). A gene or a transcript variant was considered differentially expressed if the absolute fold change (FC) was greater than or equal to two and the FDR *p*-value was less than or equal to 0.05.

### 2.4. Analysis of the Transcript Variants

We analyzed the differential expression of genes using the gene expression output of RNA sequencing analysis files. Here, all the mRNA splice variants are aggregated into one value. The differentially expressed genes were divided into three groups: upregulated (FC ≥ 2 and FDR *p*-value ≤ 0.05), downregulated (FC ≤ −2 and FDR *p*-value ≤ 0.05), and insignificant (either absolute FC < 2 and/or FDR *p*-value > 0.05). We analyzed the differential expression of transcript variants using the transcript expression output of the RNA sequencing analysis files. In contrast to the gene expression output, these files include each mRNA splice variant and its expression in the ovaries. The differentially expressed transcript variants were also divided into three groups: upregulated (FC ≥ 2 and FDR *p*-value ≤ 0.05), downregulated (FC ≤ −2 and FDR *p*-value ≤ 0.05), and insignificant (either absolute FC < 2 and/or FDR *p*-value > 0.05).

The nomenclature for the transcript variants follows the Ensembl transcript annotations, where each variant is designated first by the gene name, followed by a hyphen, and then a three-digit number, starting from 201. 201 or 202 is typically the Ensembl canonical transcript, which is the most conserved, highly expressed, and has the longest coding sequence. Those transcripts have been discussed in numerous key resources, including NCBI and UniProt. Nonetheless, the Ensembl canonical transcripts can vary from gene to gene, so it is essential to consider the official Ensembl website for further details.

### 2.5. Gene and Transcript Switching Analysis

After the data were analyzed, genes that showed no expression were extracted from the first sample of the control group. The resulting list of genes was then filtered in the following sample. After repeating the process for all samples, a list of genes with no expression in any sample was the resultant output. The non-expressed list of genes was filtered in the experimental group to obtain a list of genes switched on due to ovarian aging. The exact process was performed with both the experimental and control groups to produce a list of genes that were switched off. The protocol for transcript switching is like that of gene switching.

### 2.6. Statistical Analyses

For RNA-Seq analysis, each study group included five library samples. In the CLC GW, the ‘Differential Expression for RNA-Seq tool’ performs multi-factorial statistics on a set of expression tracks using a negative binomial generalized linear model (GLM). The final GLM fit and dispersion estimate calculate the total likelihood of the model given the data and the uncertainty of each fitted coefficient. Two statistical tests—the Wald and the Likelihood Ratio tests—use one of these values. The across-groups (ANOVA-like) comparison uses the Likelihood Ratio test.

## 3. Results

### 3.1. Changes in Aging Ovaries

**Figure 1:**
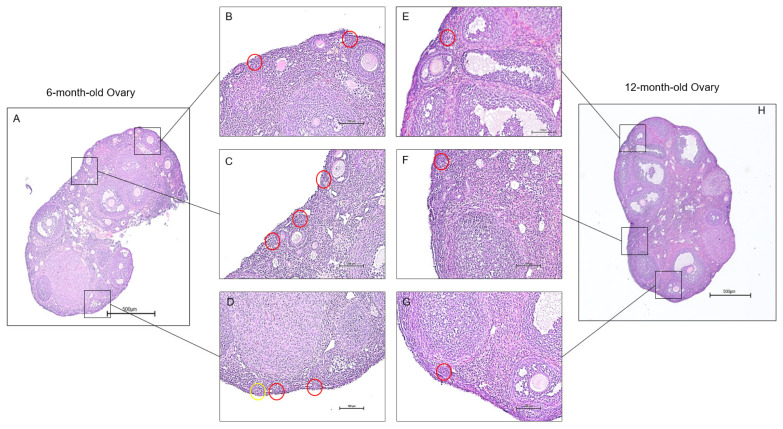
Histology of aging mouse ovaries. Ovaries from 6-month-old and 12-month-old mice were isolated for histological examination. The 6-month-old ovaries (**A**) exhibited more primordial follicles than the 12-month-old ovaries (**H**). Sections from the 6-month-old ovaries (**B**–**D**) display multiple primordial follicles. In contrast, sections of the 12-month-old ovaries showed a limited presence of primordial follicles (**E**–**G**). The red circles outline primordial follicles, and the yellow circles outline primary follicles or transitional follicles. (scale bar = 500 μm).

Continued decrease in the number of ovarian follicles and oocyte quality poses a direct link to ovarian aging [31]. To understand the molecular mechanisms that affect ovarian follicles, we focused on the expression changes of mRNA splice variants in young and old mouse ovaries. These shifts in expression provided insight into the roles of each transcript during ovarian aging. However, observing the phenotypic changes in aging ovaries precedes data analysis, as it allows the effects of senescence to become apparent. Histological sections of young and aged ovaries revealed follicular changes associated with aging (Figure 1). Since the reproductive capabilities in mice begin to deteriorate rapidly after six to nine months of age due to loss of follicle reserve, signs of infertility appear in 12-month-old mice compared to 6-month-old mice [32]. We can see this difference in the quantity and quality of the primordial follicles present in the ovaries. As a result, the 6-month-old ovary is expected to exhibit more primordial follicles than the 12-month-old ovary, and primordial follicles in the 12-month-old ovary were rarely observed.

### 3.2. Evaluation of RNA-Seq Data

After examining the phenotypic changes in the ovaries, we compared the RNA-Seq data from 6- and 12-month-old mouse ovaries and identified differentially expressed genes and transcript variants. The RNA sequencing data were filtered using a FastQC quality control test before being analyzed with CLC GW tools. Regarding per-base sequence quality, each Fastq RNA sequencing file had a Phred score greater than 30, which was consistently observed throughout all bases, with no noticeable drop-off towards the ends of the reads. The per-sequence quality scores maintained a mean Phred score greater than 30, and the per-base sequence content displayed an even base composition for every position of the read. GC content was consistent throughout all sequences, per-base N content was very low, and sequence duplication levels were low. The only concern was in the overrepresented sequences, which indicated the presence of an adapter. These adapters were eliminated through a trimming process.

**Figure 2:**
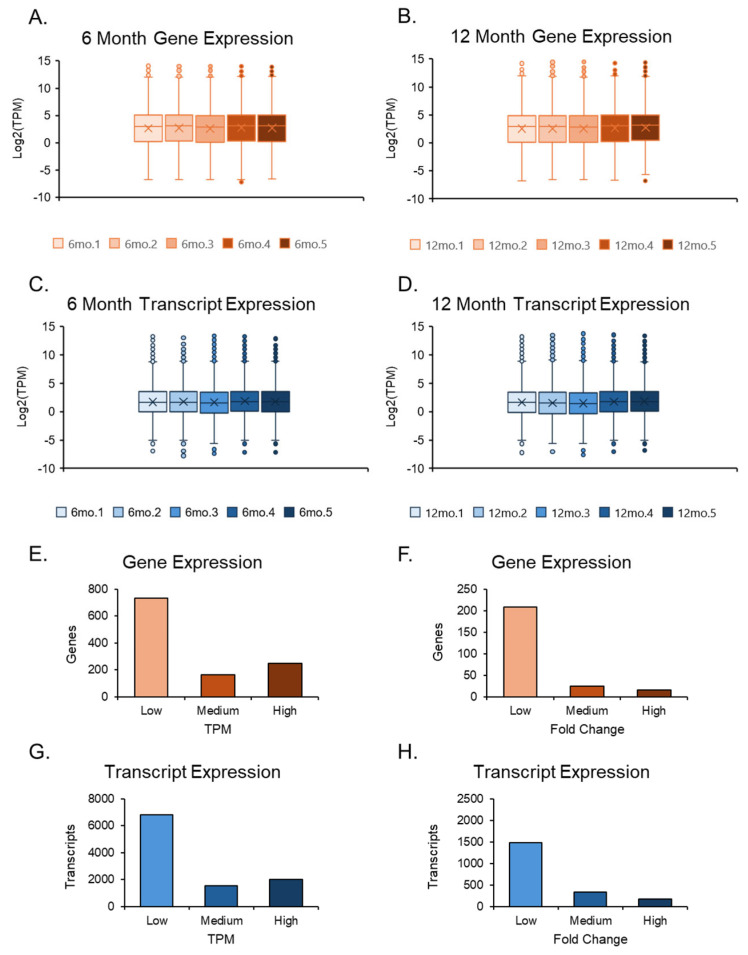
Expression plots of the overall datasets. Box and whisker plots of both 6-month and 12-month data sets were separated; their gene and transcript expression values were evaluated after normalization. The 6-month expression data (**A**,**C**) among the 5 samples were compared. The 12-month expression data (**B**,**D**) were compared in a similar manner to the 6-month data. Gene and transcript expression were further assessed by separating TPM values into three groups: low (1 < TPM ≤ 5), medium (5 < TPM ≤ 10), and high (TPM > 10) (**E**,**G**). Additionally, the same form of analysis was conducted for fold change (FC) values by separating them into three groups: low (2 < FC ≤ 5), medium (5 < FC ≤ 25), and high (FC > 25) (**F**,**H**).

The RNA sequencing data were imported into CLC GW, where adapter removal and accurate sequencing were performed after trimming the reads. RNA-seq results were cross-verified by both genomics programs and showed a promising arrangement along the first principal component axis. The two data sets, 6-month and 12-month ovaries, also displayed slight variation in their respective groups with similar averages, maxima, minima, and outliers. Still, differences were observed between the two groups in averages, maxima, and top outliers, where the transcripts were expressed at higher TPMs for gene and transcript levels in the 12-month-old group (Figure 2A–D). However, due to the number of genes compared to transcripts, the transcript expression plots had tighter averages, balanced by a larger number of outliers. The quantities between genes and transcripts were similar when examining TPMs and FCs in isolation (Figure 2E–H).

### 3.3. Differential Expression of Genes in Aging Ovaries

**Figure 3:**
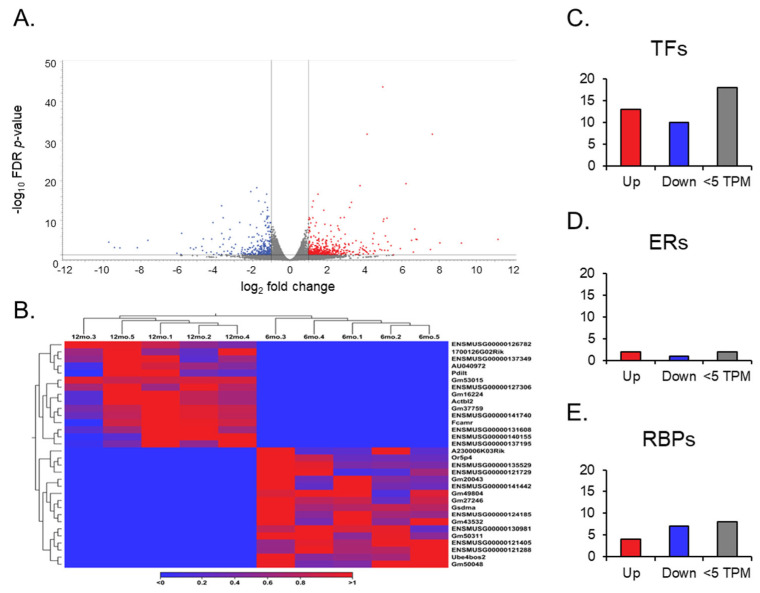
Volcano plots of gene expression, heat maps of gene switching, and bar graphs of gene groups. The volcano plot (**A**) displays all expressed genes when comparing the middle-aged and young ovaries. The genes with low fold change (FC) and high *p*-value values are faded out. The red and blue points represent differentially expressed genes: red points indicate upregulated genes, and blue points indicate downregulated genes in middle-aged ovaries compared to young ovaries. (**B**) Heat map for the expression profiles of all genes switched off and on in either young or aged mouse ovaries. The expression levels are visualized using a gradient color scheme, where the right side of the bar (red) represents high expression and the left side (blue) represents low expression relative to the data set. Significant genes from each group, including ERs, TFs, and RBPs, were categorized into upregulated (FC ≥ 2), downregulated (FC ≤ 2), and low TPM (<5) groups (**C**–**E**).

After the RNA sequencing analysis, a gene expression output was observed in the differential expression of genes in aging ovaries. Compared to the ovaries from 6-month-old young mice, the ovaries from 12-month-old middle-aged mice showed 337 differentially expressed transcript variants out of 64,470 genes compiled in the GRCm39 reference genome (Figure 3A). Of those, 182 genes were upregulated, and 155 were downregulated (absolute FC ≥ 2 and FDR *p*-value ≤ 0.05), with a TPM larger than 5.

Further analysis of expression results allowed for the observation of gene switching (Figure 3B). Genes that exhibited no expression across all data entries in one group, either at 6 months or 12 months, were filtered in the opposite group for expression to identify genes that have been switched on (no expression in the young ovaries; expression in the aged) and off (no expression in the aged ovaries; expression in the young ovaries) during the aging process in the ovary. Of the 17 genes switched off, only one, *Gm49804*, was highly expressed (TPM > 10) in the young ovary. Of the 15 genes switched on after aging, *Gm53015* was the only highly expressed (TPM > 10) in the aged ovary. In addition, we analyzed differentially expressed genes with a TPM greater than 5, and found 23 TFs, 3 ERs, and 11 RBPs (Figure 3C–E).

Out of the 23 TFs, 13 were significantly upregulated. Specifically, *Zbed6* and *Sfrp4* were noted due to their high FC (>30) and expression value (TPM > 2000), respectively. Of the 10 downregulated *TFs*, *Nr1d1*, *Osr2*, *Dbp*, and Hspa1b exhibited TPMs > 100. However, in the ERs, only two genes were significantly upregulated, and one gene was significantly downregulated. Notably, Phf20l1, which is upregulated, and *Spen*, which is downregulated, were distinguishable in this small group, as they had TPMs above 30. For RBPs, we identified four genes that were significantly upregulated. On the other hand, seven genes were downregulated, including *Spen*, *Gm49804*, and *Nynrin*, with a TPM > 30. However, no SPs from the curated list were found to be differentially expressed.

### 3.4. Differential Expression of Transcript Variants in Aging Ovaries

**Figure 4:**
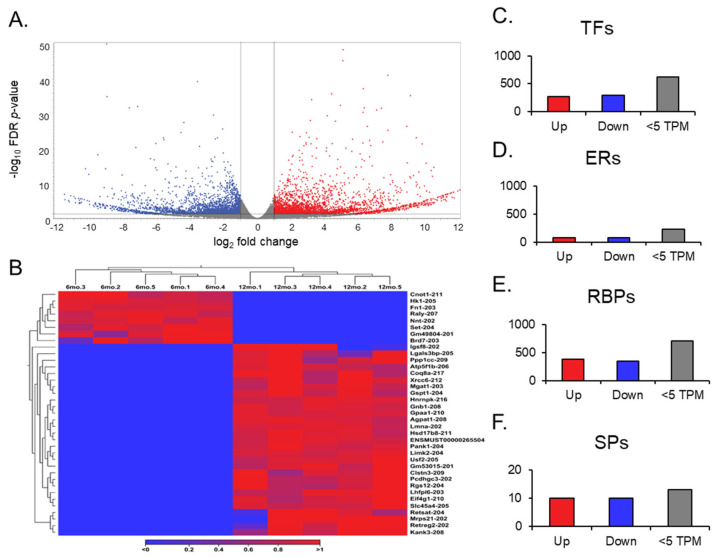
Volcano plots of transcript expression, heat maps of transcript switching, and bar graphs of transcript groups. The volcano plot (**A**) displays all expressed transcripts when comparing the middle-aged and young ovaries. The transcripts with low FC and high *p*-values are faded out. The red and blue points represent differentially expressed genes: red points indicate upregulated genes, and blue points indicate downregulated genes in middle-aged ovaries compared to young ovaries. (**B**) Heat map for the expression profiles of all transcript variants switched on and off in either young or aged mouse ovaries. The expression levels are visualized using a gradient color scheme, where the right-side color, red, represents high expression, and the left-side color, blue, represents low expression relative to the data set. Significant transcripts from each group (TFs, ERs, RBPs, and SPs) were sorted into upregulated (FC ≥ 2), downregulated (FC ≤ 2), and low TPM (<5) groups (**C**–**F**).

A transcript expression output follows a gene expression output during an RNA sequence analysis. The transcript expression produced the necessary information to understand the differential expression of splice variants in aging ovaries. When looking at each mRNA splice variant within the GRCm39 reference genome, the expression comparison between young and aged ovaries became more in-depth, as the genome compiles 260,635 total transcripts (Figure 4A). Remarkably, about 90% of the transcripts observed in each group about aging ovaries expressed more than two transcript variants based on the GRCm39.113_mRNA reference. Of the 260,635 transcripts compiled in the reference genome, 2510 were found to be differentially expressed; 1264 were upregulated (FC ≥ 2 and FDR *p*-value ≤ 0.05), and 1241 were downregulated (FC ≤ −2 and FDR *p*-value ≤ 0.05), both with a TPM > 5.

Gene switching was observed for additional analysis, but at the transcript level (Figure 4B). The transcript variants that showed no expression across all data entries in one group, either 6 months or 12 months, were filtered out in the opposite group to identify genes switched on and switched off during the aging process in the ovary. Out of the 105 transcript variants switched off after aging, 8 were highly expressed in the young ovary. Notably, *Raly-207* and *Set-204* exhibited the most significant difference, with TPMs greater than 20 before being switched off in the aged ovary. Of the 158 transcript variants switched on after aging, 30 were highly expressed in the aged ovary. Here, *Eif4g-210*, *Gnb1-208*, *Hsd17b8-211*, and *Ppp1cc-209* displayed TPMs > 30 when switched on in the aged ovary, and *Atp5f1b-206* was expressed with a TPM > 150. These significant transcript switches provide valuable insight into the mechanisms of transcriptional silencing and activation. Most transcripts that appear to be switched on and off are not the canonical variants and may result from latent transcriptional activation or silencing by long non-coding RNAs [33,34]. Looking upstream from the transcription of these specific mRNA splice variants will provide insight into what the RNAs deem necessary for the complete transcription of the ovary during the aging process.

We observed different groups of differentially expressed transcripts with TPM values above 5 (Figure 4C–F). We identified 149 ERs, 569 TFs, 728 RBPs, and 10 SPs. Among the TFs, 272 transcripts were identified as being significantly upregulated. *Sfrp4-204*, *Tsc22d1-214*, and *Nfe2l1-212* were notable transcripts within the upregulated group due to their simultaneous features of a high TPM (>100) and high FC (>10). Although the other 297 significantly downregulated group of transcripts did not exhibit the same magnitude of expression as their upregulated counterparts, *Raly-207* and *Set-204* from the transcript switching analysis reappeared as notable transcripts due to their TPMs and FCs compared to other downregulated transcripts.

For the group of ERs, 72 were significantly upregulated, and 77 were significantly downregulated. Within the upregulated group, *Ppm1g-205* and *Dek-204* stood out due to their FCs (>1000), while *Ncl-205* and *Ogt-203* also stood out due to their high TPM values (>40). *Usp3-211* remained in the downregulated group with an FC above 1000, and *Ywhaz-204* had an expression value above 100 TPMs. To analyze the RBPs, we followed a similar guideline to that of the TFs, due to their comparable transcript quantities. A total of 379 RBP transcripts were detected as significantly upregulated, and 349 were detected as significantly downregulated. *Actg1-204*, *Atp5f1b-206*, *Rplp2-203*, *Rack1-202*, *Psap-202*, *Eef1g-205* are noted for their high TPMs (>100) and high FCs (>10) in the upregulated group. *Rps3-205* and *Rpl13a-214*, on the other hand, are regarded as having the same quantitative measures, but in the downregulated group. Finally, the SPs only contained 10 significantly upregulated transcripts and 10 significantly downregulated transcripts. Of the 10 upregulated transcripts, *Slu7-206* stood out for its large FC (>1000), but *Sf3b2-208* and *Snrnp70-209* are notable for their high expression values (TPM >50). The 10 downregulated transcripts did not yield impressive quantitative results; however, *Sf1-203* was the most downregulated of the group.

### 3.5. Correlation of Differentially Expressed Genes and Transcript Variants

**Figure 5:**
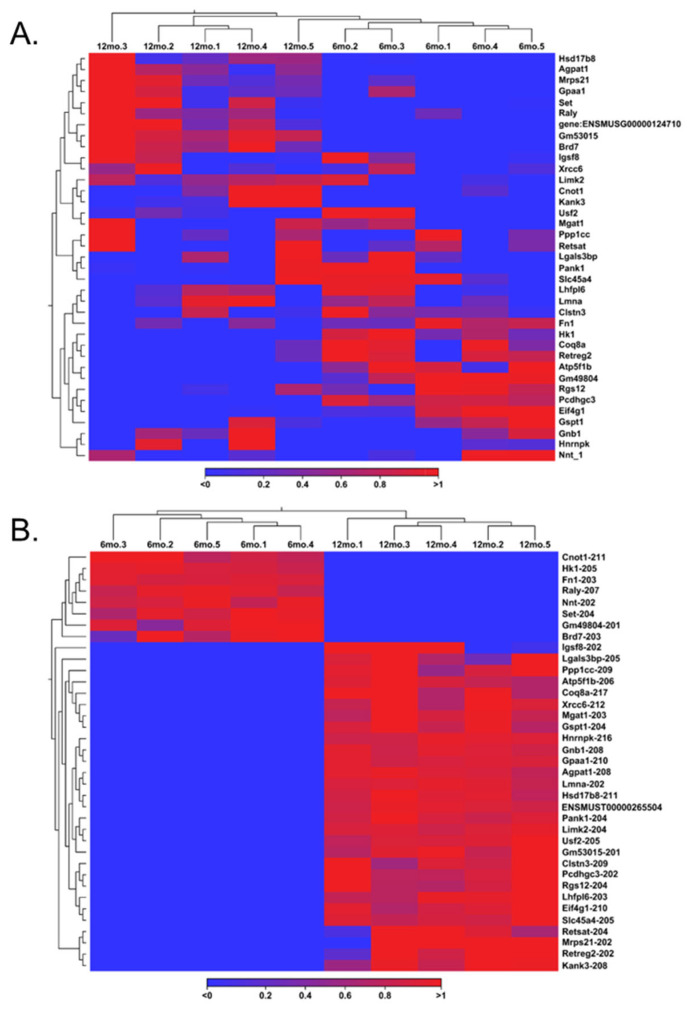
Heat maps representing mRNA transcript switching in aged ovaries. Transcripts that participated in transcript switching were filtered for gene expression analysis to identify the genes from which the transcript originated (**A**). Genes that participated in gene switching were identified and filtered for transcript expression analysis to identify the transcripts related to those genes (**B**).

Although the same genes and their corresponding transcripts were present in the GRCm39 reference genome, further analysis demonstrated a notable discrepancy between gene expression and transcript expression analysis. Discrepancies in gene and transcript switching follow the logic that multiple splice variants within one gene identifier exhibit different expression levels. Hence, a record of each splice variant of the original 337 significant genes was maintained, allowing for the observation of such discordances. This resulted in 1285 transcript variants, where only 241 were statistically significant. For confirmation, these 241 significant variants were the only variants to match the original 2505 significant variants from the transcript expression output. Of these 241 significant variants extracted from gene expression, 125 were upregulated (FC ≥ 2 and FDR *p*-value ≤ 0.05), and 116 were downregulated (FC ≤ −2 and FDR *p*-value ≤ 0.05), both with a TPM value greater than 5. By taking the genes that had switched on and off in the aging ovary and selecting their respective transcripts, a heat map was created to display the genes involved in gene switching at the gene level (Figure 5A). This process was performed for the transcripts involved in transcript switching by creating a heat map of their respective genes to illustrate transcript switching at the gene level (Figure 5B).

**Figure 6:**
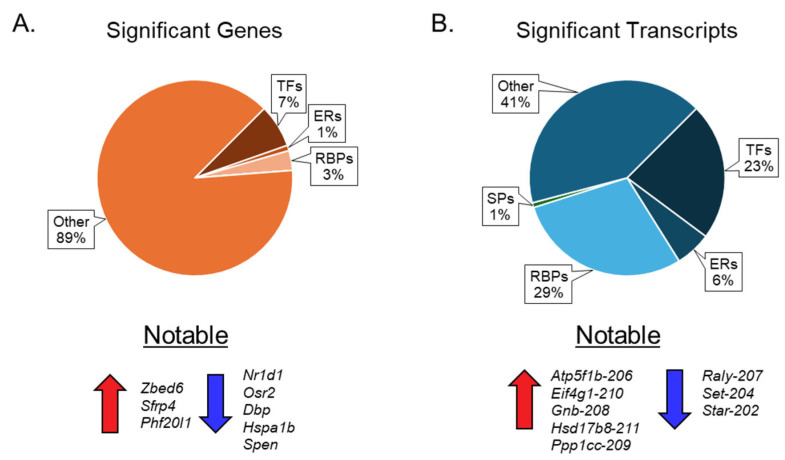
Pie chart presentation of differentially expressed genes and transcripts. The first pie chart (**A**) illustrates the percentage distribution of differentially expressed genes across various groups of epigenetic regulators (ERs), transcription factors (TFs), RNA-binding proteins (RBPs), and spliceosome components (SPs). Below the first pie chart are the notable upregulated and downregulated genes. The second pie chart (**B**) depicts the percentage distribution of significant transcription factors within the aforementioned groups of ERs, TFs, RBPs, and SPs. Below the second pie chart (**B**) are the notable upregulated and downregulated mRNA transcript variants.

Our results show that ovarian aging alters the expression of genes encoding ERs, TFs, and RBPs, but not SPs (Figure 6A). We also observed that changes in mRNA transcript variants encoding ERs, TFs, RBPs, and SPs were significantly higher than those of the corresponding genes (Figure 6B). We detected that 7% of TFs, 1% of ERs, and 3% of RBPs were differentially expressed at the gene level (Figure 6A), which increased to 23% of TFs, 6% of ERs, 28% of RBPs, and 1% of SPs at the transcript variant level (Figure 6B).

### 3.6. Mechanisms Underlying Altered Transcript Variants in Aging Ovaries

**Figure 7:**
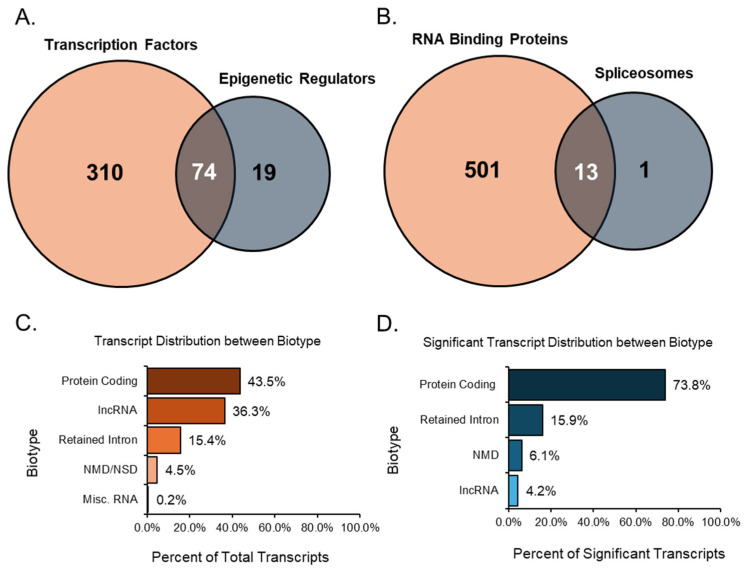
Venn diagrams of transcript groups and bar graphs of transcript biotypes. The first Venn diagram identifies significant (TPM > 10, absolute FC ≥ 2, and FDR *p*-value ≤ 0.05) transcript factors and epigenetic regulators (**A**) and highlights the transcripts that both groups shared. Similarly, the second Venn diagram identifies significant RBPs and transcripts associated with SPs (**B**), while also highlighting the transcripts present in both groups. All expressed transcript variants from the data were categorized by their biotype (**C**), giving a percentage distribution of all groups. Significant transcripts from the data were categorized by their biotype (**D**), resulting in a percentage of distribution in resultant groups.

After analyzing each group individually, we examined the relationships between related groups, such as TFs compared with ERs and RBPs compared with SPs. Overlaps were detected in the comparisons mentioned, which helped identify transcripts that play multiple essential roles. When comparing TFs to ERs, there were many overlaps between the two groups, as many transcripts share an upstream role in transcription and epigenetics (Figure 7A). Of these overlapped transcripts, four were notable due to their relationship to the ovary and high expression values when differentially expressed. For instance, the transcript *Npm1-203* had a high TPM value (TPM > 50) and a remarkably downregulated FC (<−20). An overexpression of NPM1 is linked to unsuitable outcomes for women with ovarian cancer [35]. *Mta1-202* was also a transcript that was highly downregulated (FC < −10) and expressed at a high level (TPM > 40). MTA1 is associated with advanced and metastatic ovarian cancer tissue [36]. In contrast, the transcript *Trim28-203* originated from a gene recently identified as regulating granulosa cell senescence [37]. Here, our analysis indicated that the transcript was expressed at a high TPM (>40) and upregulated heavily (FC > 10). The final transcript, *Ube2n-203*, was also highly upregulated (FC > 5) and expressed at a high level (TPM > 40). The gene UBE2N was found to be key in regulating paclitaxel resistance in ovarian cancer cells [38].

Significant transcripts associated with RNA binding proteins and spliceosomes were compared, and we found 13 unique transcripts that overlapped between these two groups (Figure 7B). Of these 13 transcripts, *Rbm25*, a known global splicing factor, appeared as two splice variants (−206 and −208). Both variants were upregulated, displaying high TPMs ≥ 10 and FCs ≥ 5. Similarly, *Sf3b1-203*, a disruptive splicing factor, was upregulated in both groups with high TPM and FC values. In contrast, *Cd2bp2-201* was a variant formed using alternative start sites and was highly downregulated in both groups. Although not the full-length canonical variant of the CD2BP2 line, *Cd2bp2-201* still encoded the same protein and was the only significant variant identified during analysis.

Most of the expressed, annotated transcript variants detected in the study were of the protein-coding biotype. These protein-coding transcript variants contain an open reading frame (ORF) that can be translated into a protein, accounting for 35.5% (45,561 transcripts) of all expressed transcripts (Figure 7C). In addition, 7.9% (10,229 transcripts) of all expressed transcripts were alternative splice variants of protein-coding transcripts without a defined coding sequence. The protein-coding transcripts comprised 43.5% (55,790 transcripts) of all transcripts. Following the protein-coding biotype, long non-coding RNA (lncRNA) comprised 36.3% (46,548 transcripts) of all expressed transcripts. These lncRNAs did not contain meaningful open-reading frames but were longer than 200 base pairs. Another notable biotype included those involved in decay processes such as nonsense-mediated decay (NMD) and non-stop decay (NSD).

The transcripts classified as nonsense-mediated decay had a premature stop codon, which is most likely a result of targeted degradation. For Ensembl, nonsense-mediated decay prediction happened when an in-frame termination codon was found more than 50 base pairs upstream from the final splice junction. Transcripts related to decay made up 4.5% (5809 transcripts) of the expressed transcripts in the study. Intron retention was another alternative splicing outcome, where the intron was kept rather than spliced out. Transcripts categorized as retained introns were splice variants with similar intronic sequences to those of other transcripts produced from the same gene locus; these transcripts comprised approximately 15.4% (19,807 transcripts) of all expressed transcripts. Finally, miscellaneous RNA comprise a combination of non-coding RNAs that cannot be classified, RNA components of the ribosome, snRNAs, snoRNAs, miRNAs, and mitochondrial RNAs, accounting for 0.2% (266 transcripts) of all expressed transcripts.

After filtering significant transcripts from the expression data with a minimum TPM threshold of 5, an absolute FC of at least 2, and an FDR *p*-value of 0.05 or lower, we identified the biotypes of all resultant transcripts (Figure 7D). Here, protein-coding transcripts, including those without a defined coding sequence, accounted for 73.8% (1853 transcripts) of the significant transcripts. Interestingly, the next-largest group of biotypes was the retained intron transcripts, which comprised 15.9% (400 transcripts) of the differentially expressed transcripts. NSD transcripts were not detected as significant, so only NMD was recorded. These decay transcripts comprised 6.1% (152 transcripts) of substantial transcripts. Despite being the second-largest biotype in the total transcript analysis, lncRNA accounted for 4.2% (105 transcripts) of the significant transcripts. The shift from lncRNA to more protein-coding transcripts may give insight into the mechanisms involved in the ovarian aging process.

## 4. Discussion

Nearly half of the global population experiences a range of endocrine, metabolic, and neurological disorders as they enter the later stages of life, with a significant portion of these issues stemming from ovarian aging [39]. The ovaries are among the first organs to show functional decline, particularly in reproductive capacity [40]. This has profound implications for women’s health and well-being. As the ovaries age, they undergo a series of complex changes that impact hormonal balance, tissue integrity, cellular function, and gene regulation [5]. Despite recently developed therapies aimed at mitigating the symptoms of ovarian aging, such as hormone replacement therapies (HRTs), these treatments often come with significant side effects, raising concerns about their safety and efficacy for long-term use [41]. A comprehensive understanding of the cellular and molecular mechanisms underpinning ovarian aging is essential for devising targeted interventions to enhance women’s health outcomes.

Various molecular hallmarks of aging contribute to the decline in ovarian function [42]. These hallmarks include genomic instability, which refers to the increased likelihood of mutations; telomere attrition, which is the progressive shortening of protective chromosome ends; epigenetic alterations that affect gene expression without changing the DNA sequence; loss of proteostasis, which disrupts the balance of protein synthesis and degradation; dysregulation of nutrient sensing pathways, mitochondrial dysfunction that affects energy production; cellular senescence, which is the process by which cells lose their ability to divide; stem cell exhaustion, leading to a reduced capacity for tissue regeneration; and altered intercellular communication, which can disrupt the signaling pathways necessary for maintaining ovarian health [43,44,45,46,47,48,49,50,51]. Using bulk RNA sequencing, differentially expressed genes in aging mammalian ovaries can be identified. We have conducted an in-depth analysis of gene regulation at the transcript level, providing insight into the actual changes in the ovarian transcriptome. Our analysis has revealed significant differences in the expression of various transcript variants within the ovary; this phenomenon is not exclusive to rodents but is likely also present in humans and primates. Furthermore, the expression of these transcript variants is linked to upstream regulatory mechanisms involving epigenetic and transcription factors. Meanwhile, downstream processes, such as RNA modifications mediated by RBPs and SPs, play a crucial role in shaping the final output of gene expression.

Observing significant differences in aging ovaries can be challenging, as our expression plots revealed minimal variation across different age groups. However, histological analysis revealed a nuanced picture, demonstrating notable phenotypic changes. Specifically, the ovaries of young mice are typically characterized by a substantially higher number of primordial follicles than those of middle-aged mice. This discrepancy in follicular quantity is likely a result of the transcriptomic changes that occur as ovaries age, which can alter the molecular landscape of ovarian function and development. A more thorough examination of the transcripts involved can yield a more accurate depiction of the aging process, due to the enhanced quantity and resolution that modern transcriptomic analyses provide. Given the focus of the current study on quantitatively assessing specific gene groups about aging, the choice of software for data analysis is paramount. CLC’s genomics software stands out for its use of a specific algorithm, the expectation-maximum algorithm, which has been demonstrated to perform consistently with high accuracy. When benchmarked against other popular aligners, such as STAR and NOVOALIGN, CLC has been recognized for its performance, especially when analyzing data at the ’junction level’ [52]. This level of precision is crucial for understanding the complexities associated with gene expression in aging ovaries. Consequently, this study predominantly employs CLC, as it demonstrates top performance across all datasets, except for those that are the least complex, where complexity is determined by the difficulty of alignment in a specific region.

Gene expression analysis was initially done to extract as much information as possible, despite the challenge of aggregating all transcripts into a single gene identifier. Out of the 64,470 genes in the reference genome, 38,159 were expressed. However, using a stringent filtering process, we discovered that only 337 met our established criteria denoting statistical significance. These criteria included a minimum TPM ≥ 5, an absolute FC of ≥ 2, and an FDR *p*-value ≤ 0.05. Among the different groups analyzed, we found that TFs yielded 24 significant genes, approximately 7% of the total significant genes identified in this study. In stark contrast, ERs identified only three significant genes, representing 0.9% of the significant gene pool. It is essential to note that the overlap between TFs and ERs resulted in a combined 24 distinct genes, underscoring the interconnected nature of these regulatory mechanisms. This suggests that approximately 7% of the genes highlighted in this study play some role in transcriptional regulation or epigenetic modifications. The group comprising RBPs and SPs revealed only a modest enhancement over the contributions of TFs and ERs. A total of only 11 significant genes were identified from the RBPs, while SPs did not contribute any significant genes to the analysis. This group accounted for approximately 3% of the significant genes identified in this study. The gene switching analysis, aimed at identifying genes with completely altered expression patterns, identified a limited number of genes. Among these, only two genes exhibited a TPM ≥ 10: *Gm53015*, which was found to be upregulated in the aged ovary, and *Gm49804*, which was found to be downregulated. Despite the seemingly low involvement of each group in the overall gene expression analysis, several notable genes emerged due to their remarkably high expression levels and significant FCs in aged ovaries. Specifically, the genes *Zbed6*, *Sfrp4*, and *Phf20l1* demonstrated substantial upregulation, indicating their potential importance in ovarian aging. Conversely, genes such as *Nr1d1*, *Osr2*, *Dbp*, *Hspa1b*, and *Spen* exhibited significant FCs and high TPM values despite being downregulated. These findings underscore the complexity of gene expression dynamics and highlight key players that may be pivotal in ovarian aging.

A more detailed transcript expression analysis was conducted following the gene expression analysis, yielding more promising outcomes. Out of the 260,635 transcripts available in the reference genome, 128,220 were expressed, resulting in the detection of 2510 significant transcripts (TPM ≥ 5, absolute FC ≥ 2, and FDR *p*-value ≤ 0.05). At the transcript level, 569 significant transcripts were identified, representing approximately 23% of the significant transcripts detected in this study. Notably, the number of significant ERs increased to approximately 6% for transcripts, with 149 detected. Following the removal of overlapping transcripts and the subsequent compilation of a list of TFs and ERs, 604 significant transcripts were identified. This indicated that approximately 24% of the significant transcripts identified in this study were associated with ERs or TFs, suggesting their potential in regulating transcriptional processes or epigenetic modifications, an impressive enhancement compared to the initial data derived solely from gene expression analyses. For RBPs, a total of 728 significant transcripts were identified, which accounted for 29% of the overall significant transcripts in the study. The SPs also showed a notable increase, with 20 significant transcripts detected. Although SPs only accounted for approximately 0.8% of the significant transcripts, the opportunity to investigate these 20 specific transcripts in relation to their roles in ovarian aging is considerably more feasible than conducting a broad gene-level analysis, particularly since no SPs emerged as significant at the gene level. When compiled, RBPs and SPs together constitute approximately 29% of the significant transcripts identified. This suggests that 731 transcripts may possess the capacity to influence downstream biological processes through the modifications they impose on RNA, especially in the context of ovarian aging. Switching analysis proved to be more effective than the gene switching analysis, revealing 30 transcripts that were activated in the aged ovary, each exhibiting a TPM greater than 10, alongside 8 transcripts that were switched off. Each of these highly expressed transcripts exhibited overlap with at least one of the four groups used in the expression analysis. Specific transcripts such as *Atp5f1b-206*, *Eif4g1-210*, *Gnb1-208*, *Hsd17b8-211*, and *Ppp1cc-209* are recognized for their high expression when switched on, while *Raly-207* and *Set-204* are highlighted for their high expression despite downregulation in the aged ovary. This nuanced understanding of transcript dynamics in the context of ovarian aging presents a valuable opportunity for further exploration.

Despite the vast number of long non-coding RNAs present within the dataset, a significant majority of the identified transcripts were classified as protein-coding. These transcripts contain ORFs that enable protein translation, ultimately leading to the expression of specific biological functions [53]. Although many transcript variants code for the same protein, some may diverge and encode proteins with differences in sequence, length, or functional domains (e.g., enzymatic activity, binding capability, etc.) [51]. This variability in coding sequences among transcripts emphasizes the critical need to examine individual transcripts and their unique contributions to the transcriptome. Generalizing the transcript variants to their gene identifiers may mask their true functional significance [54]. Accordingly, gene and transcript expression analysis often yield divergent outcomes, reflecting the inherent differences in the methodologies employed. Gene expression analysis provides a broader and more rapid overview of the dataset, effectively summarizing the expression levels of the aggregated transcripts. However, it has a minor caveat in that one cannot determine whether the canonical transcript directly leads to the mechanistic function or if it is a splice variant that is masked by its canonical counterpart. Conversely, the more detailed approach of transcript expression analysis permits a more thorough evaluation of individual transcripts, allowing a deeper understanding of expression patterns. By examining transcripts in isolation, more expression data can be extracted, and more accurate representations of gene expression values can be derived. It is crucial to recognize that the expression of a single transcript can neither be reliably used to evaluate the expression of a gene, nor can the reverse be applied. Gene and transcript expression analysis each have their advantages and disadvantages. However, this study prioritizes the value of transcript expression in understanding the complexities of aging ovaries.

Throughout the study, transcripts from the genes *Gnb1*, *Star*, and *Sfrp4* were recognized for their dramatic shifts in expression in the aged ovary. A recent study showed that *GNB1* is involved in PCOS [55]. Our analysis demonstrates that *Gnb1-208* was switched on in the aged ovary with a high expression value (TPM > 10). According to the Ensembl database, *Gnb1-208* codes for the same protein as its canonical counterpart, sharing functional relevance; however, it was expressed differently, as *Gnb1-203*, the canonical transcript, was downregulated. The protein *Gnb1-208* acts as a modulator or a transducer in transmembrane signaling systems related to the growth and differentiation of follicular cells affected by PCOS (Supplemental Table S1A) [55]. Thus, altering the expression of *Gnb1-208* may delay or prevent PCOS, ultimately halting a symptom of ovarian aging.

*Star* has been demonstrated to be an essential component in steroidogenesis during follicular maturation [56]. Our analysis identified the splice variant *Star-202* as a significantly downregulated transcript in the entire aged ovary. Notably, *Star-202* encodes a protein different from its canonical counterpart, *Star-201*, despite sharing a similar effect on steroid production. Studies show that *Star-201* is essential for the biosynthesis of steroid hormones in theca cells, whereas *Star-202* has little to no information on the function of the protein it encodes [57]. However, splice mutations in the human *STAR* gene are suspected to play a role in gonadal failure due to premature loss of ovarian follicles, potentially leading to PCOS [58]. Since the canonical transcript’s expression did not change in the aged ovary, but the splice variant significantly changed, manipulating the expression of the splice variant, *Star-202*, could be investigated as a potential strategy to improve the follicular reserve (Supplemental Table S1B).

Finally, secreted fizzled-related sequence protein, *SFRP4*, has been characterized as a negative regulator of ovarian follicle development, limiting the ovarian reserve and reducing female fertility [59]. Unlike the *Star* transcripts, the variant *Sfrp4-204* was highly upregulated in aging ovaries (Supplemental Table S1A). *Sfrp4-204* does not produce the same protein as its canonical counterpart, *Sfrp4-203*, indicating that these two variants fulfill distinct biological roles. However, both transcripts were upregulated in our analysis, and lower expression of *Sfrp4* can increase the ovarian reserve for female fertility. This is because soluble frizzled-related proteins act as modulators of the WNT signaling pathway by directly interacting with it, ultimately regulating cell growth and differentiation of follicular cells in the ovary [60]. These transcripts associated with ovarian follicle dynamics may serve as critical molecular indicators that could delay the detrimental effects of ovarian aging through targeted expression modifications. Targeting the functional decline of the aging ovary may represent an optimal strategy for mitigating the impact of ovarian senescence, which leads to the stagnation of aging ovaries. Modulating transcript expressions could provide a foundational approach for advancing this field of research.

## 5. Conclusions

We analyzed the transcriptomes of young and aged mouse ovaries to identify changes in gene expression associated with ovarian aging. In addition to the conventional ‘one gene, one mRNA’ strategy, we have also analyzed the changes in mRNA transcript variants in aging ovaries. Our analyses identified remarkable changes in transcript variants during ovarian aging in mice. To understand the mechanisms underlying altered transcript variants, we selectively analyzed key regulatory transcripts of ERs, TFs, RBPs, and SPs. We identified a significant number of differentially expressed regulators that may have contributed to the expression of various transcript types during ovarian aging. However, the differential expression of the regulators needs to be further verified, and future loss-of-function or gain-of-function studies are required to validate the functional role of DETVs in ovarian aging. The implications of our findings on transcript variation are profound, as they may directly influence the quantity and quality of oogenesis and steroidogenesis, thereby affecting female fertility and women’s health. Moreover, identifying these transcripts and their presumed biological functions provides valuable insights into the molecular underpinnings of ovarian aging.

## Data Availability

The RNA-Seq data were downloaded from the Sequence Read Archive (SRA) at NCBI (PRJNA1195555). Further inquiries can be directed to the corresponding author(s).

## Supplementary Materials

The following supporting information can be downloaded at: https://www.mdpi.com/article/10.3390/cells14130996/s1, Table S1. Differentially Expressed Transcript Variants in 12-month-old ovaries.
