# Supplementary material for: Aging Alters mRNA Processing in the Mouse Ovary
Source: Cells. 2025 Jun 30;14(13):996. doi: 10.3390/cells14130996 (PMC12249410; doi:10.3390/cells14130996)
Supplement: Supplementary file 1 [file cells-14-00996-s001.zip › cells-3621466-supplementary.pdf]

Supplementary Table S1. Differentially Expressed Transcript Variants in 12-month-old ovaries

Supplementary Table S1A. Top 10 upregulated transcript variants in 12-month-old ovaries.

Name	Max Group Mean	Fold Change	FDR p-value	Coded Potential Protein Function	ENSEMBL ID
Rbm39-217	92.70	16,888.19	1.33E-09	Pre-mRNA splicing factor/ Transcriptional coactivator of ESR2	ENSMUST00000146297
Gnb1-208	48.89	9,769.03	1.31E-10	Modulator/transducer in transmembrane signaling systems related to follicular cells	ENSMUST00000176637
Hnrnpk-216	22.41	4,147.61	1.20E-08	Regulation of TP53 in response to DNA damage is common in ovarian carcinomas	ENSMUST00000177019
Actg1-204	506.55	3,561.24	2.73E-103	Promotes granulosa cell migration	ENSMUST00000106215
Inhba-202	21.82	2,244.83	9.67E-7	Inhibits the secretion of follicular hormone by the pituitary gland	ENSMUST00000164993
Grk2-201	20.06	1,534.86	2.16E-15	Phosphorylates agonist-occupied G protein-coupled receptors	ENSMUST00000025791
Hnrnpf-210	76.32	106.59	1.93E-18	Plays a role in the regulation of alternative splicing events	ENSMUST00000180341
Sfrp4-204	524.96	81.73	5.63E-38	Modulator of Wnt pathway, regulating growth & differentiation of follicular cells	ENSMUST00000222992
Ptpa-202	60.02	77.50	1.06E-17	Primordial follicle maintenance	ENSMUST00000113601
Fads1-202	70.52	37.13	5.06E-11	Polyunsaturated fatty acid biosynthesis is linked to reproductive hormone synthesis	ENSMUST00000235160

Supplementary Table S1B. Top 10 downregulated transcript variants in 12-month-old ovaries.

Name	Max Group Mean	Fold Change	FDR p-value	Coded Potential Protein Function	ENSEMBL ID
Fn1-203	15.52	-8,237.19	6.23E-10	Strengths of ovarian extracellular matrix via integrin receptor signaling	ENSMUST00000186129
Entpd4-201	23.85	-2,972.44	4.10E-06	Regulates PI3K/Akt pathway by hydrolyzing UDP to accelerate glycosylation	ENSMUST00000064831
Epb41l2-201	8.63	-2,551.22	6.88E-06	Oncogenes in ovarian cancers	ENSMUST00000053748
Ambra1-205	7.48	-2,327.64	3.24E-07	Activator of mitophagy alterations observed in age-related degenerative disorders	ENSMUST00000111317
Hk1-205	10.18	-2,027.03	5.63E-07	The first rate-limiting enzyme in glycolysis of ovarian cancer progression	ENSMUST00000130422
Usp3-211	12.66	-1,650.05	2.95E-05	Catalyzes ubiquitylation of K63 ubiquitin chain, recruits BRCA1, tumor suppressor gene	ENSMUST00000174387
Pum1-203	6.09	-1,626.90	5.43E-05	RNA-binding protein acts as a post-transcriptional repressor, regulating granulosa cell function	ENSMUST00000097864
Flna-201	56.40	-522.08	8.45E-51	Asymmetric division during meiotic maturation in oocytes	ENSMUST00000033699
Ube2i-233	11.12	-285.72	8.33E-05	Post-transcriptional modification SUMOylating in oocytes	ENSMUST00000249701
Star-202	365.28	-77.17	3.29E-89	Steroidogenesis in follicular maturation	ENSMUST00000210565
